# Steady-state magnetic resonance angiography of the thoracic vasculature in congenital heart disease using a blood pool contrast agent: evaluation of two different techniques

**DOI:** 10.1186/1532-429X-14-S1-P120

**Published:** 2012-02-01

**Authors:** Jennifer A Febbo, Mauricio S Galizia, Andrada R Popescu, Xiaoming Bi, Jeremy Collins, Michael Markl, Robert R Edelman, James Carr

**Affiliations:** 1 Radiology, Northwestern University, Chicago, IL, USA; 2 Cardiovascular MR R&D, Siemens Healthcare, Chicago, IL, USA; 3 Radiology, NorthShore University HealthSystem, Evanston, IL, USA; 4 Medical Imaging, Children's Memorial Hospital, Chicago, IL, USA

## Background

Although contrast-enhanced first-pass MRA (FP-MRA) is frequently used to visualize the vasculature, it may not be suitable for the assessment of congenital heart disease. Blood-pool contrast agents remain within the intravascular space for several hours, allowing vessels to be imaged without relying on accurate contrast bolus timing. The purpose of this study is to compare steady-state magnetic resonance angiography (SS-MRA) following injection of a blood-pool contras agent to first-pass MR angiography (FP-MRA) in adults with congenital heart disease.

## Methods

23 adult patients (15 men, 8 women) with congenital heart disease underwent MRA on a 1.5 T scanner (Magnetom Aera and Avanto; Siemens Medical Solutions). The MRA protocol consisted of FP-MRA followed by SS-MRA after intravenous injection of gadofosveset tridosium (Ablavar, Lantheus Medical Imaging). FP-MRA consisted of a breath-held ECG-gated FLASH acquisition in a coronal orientation with the following imaging parameters: TR/TE: 2.8/1.0, flip angle 25o, FOV 343x500 mm, matrix 264x512, slice thickness 1.5 mm, voxel size 1.3 x 1.0 x 1.0 mm, GRAPPA x 2, 20 second acquisition. 0.03 mmol/kg of gadofosveset was injected intravenously at 1cc/sec in an antecubital vein. Contrast bolus timing was achieved using care bolus technique. SS-MRA consisted of free-breathing ECG-gated IR-FLASH and IR-SSFP in a coronal orientation. IR-FLASH had the following parameters: TR/TE/TI: 3.5/1.5/260, flip angle 18o, and IR-SSFP: TR/TE/TI: 3.3/1.5/260, flip angle 70o. Both sequences had FOV 326x380, matrix 440x512, slice thickness 1.5 mm, voxel 0.7 x 0.7 x 1.0 mm, GRAPPA x 2, and 3 minute acquisition. Respiratory gating was achieved using a navigator acquisition with an average acceptance window of 35%. For quantitative analysis, orthogonal dimensions of the thoracic aorta were measured at several aortic locations. For qualitative analysis, two independent reviewers evaluated both FP-MRA and SS-MRA images separately. The following vessels were scored on an image quality scale of 1-4: aortic root, ascending aorta, aortic arch, descending aorta, main pulmonary trunk, right pulmonary artery, left pulmonary artery, superior vena cava, inferior vena cava. The presence or absence of pathology was noted and given a confidence score of 1-4.

## Results

**Figure 1:**
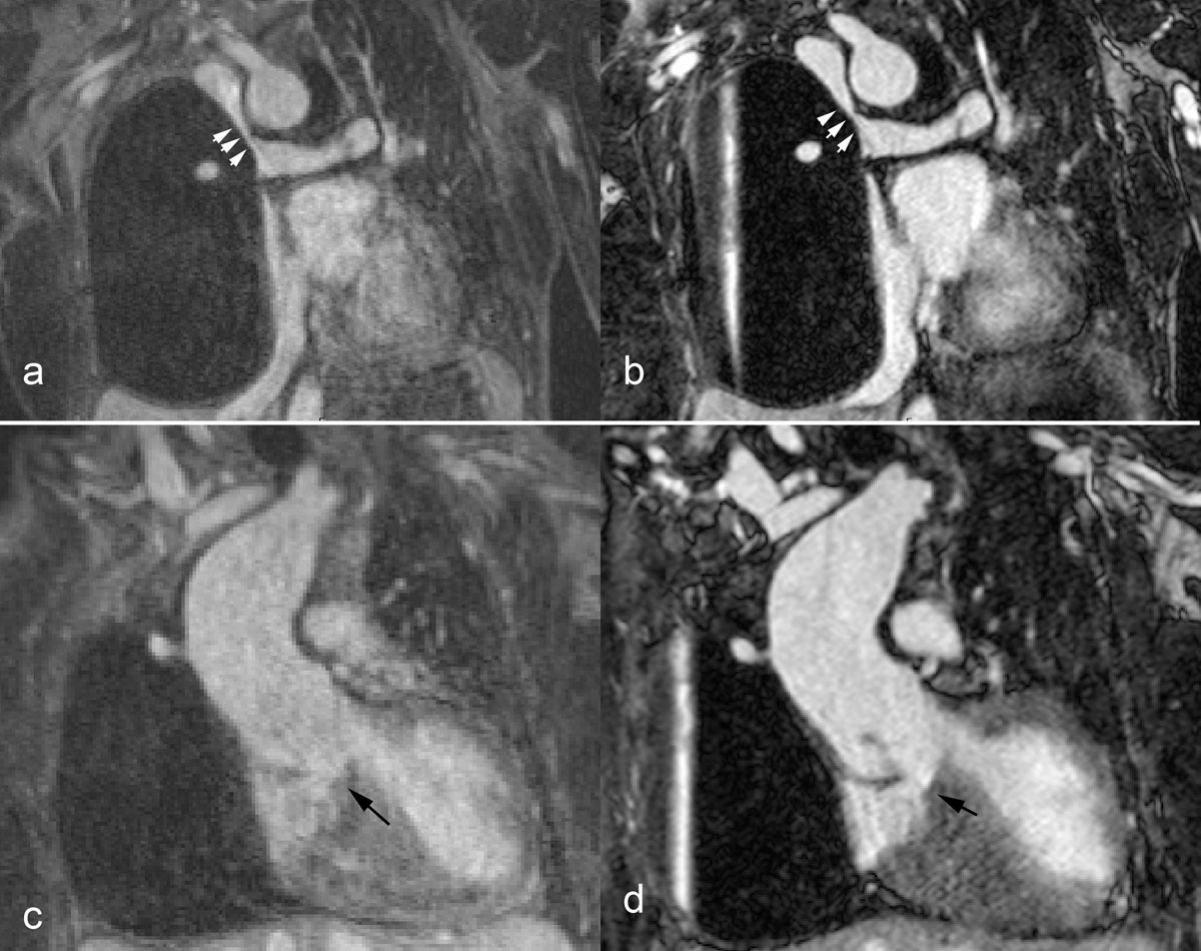
FP-MRA (a, c) and SS-MRA (b, d) of a 24-year-old woman with a diagnosis of congenital pulmonary atresia and ventricular septal defect. She had been treated with an ascending aorta to right pulmonary artery conduit and a Glenn shunt. (a, b) Glenn shunt between the superior vena cava and the right pulmonary artery (white arrowheads). (c, d) Ventricular septal defect (black arrows). Of note, the patient has a right mediastinal cystic structure causing external compression of the right lung.

There was no significant difference in aortic dimensions at all anatomic locations between FP-MRA and SS-MRA. Image quality scores were higher for SS-MRA compared to FP-MRA (Figure 1). More pathological abnormalities were seen with SS-MRA than FP-MRA.

## Conclusions

SS-MRA using either IR-FLASH or IR-SSFP was comparable to FP-MRA for measurement of aortic dimensions, but it was better to assess congenital heart diseases. SS-MRA may be a useful adjunct to FP-MRA in cases of bolus mistiming, or may potentially replace FP-MRA, thereby simplifying MRA assessment of the heart and thoracic vasculature.

## Funding

None.

